# Progress

**Published:** 1869-11

**Authors:** 


### PROGRESS.

THE formation of the "Southern Dental Association" was a long step in the right direction. That the members composing it, in spite of bad advice to the contrary, had the good taste to organize it as an auxiliary to the "American Dental Association," and thereby place themselves in active co-operation with the progressive portion of the profession throughout the nation, is very gratifying, and worthy of cordial and hearty commendation. Great good will be done by it. State and local societies will spring up all over the South, as is already evidenced by the formation of the State Society of Alabama, and others. We hope soon to record in our monthly bulletin, a society for every state and every city in the South, and to have our pages enriched with their contributions and proceedings. W.

